# [Retracted] Protective effect 3,4-dihydroxyphenylethanol in subarachnoid hemorrhage provoked oxidative neuropathy

**DOI:** 10.3892/etm.2025.12923

**Published:** 2025-07-14

**Authors:** Yu-Wen Zhong, Juan Wu, Hua-Long Hu, Wei-Xin Li, Yong Zhong

Exp Ther Med 12:1908-1914, 2016; DOI: 10.3892/etm.2016.3526

Following the publication of this paper, it was drawn to the Editor’s attention by a concerned reader that there appeared to be several potential splicing events/breaks in the continuity of the IL-6 and IL-1β bands shown in the western blot data in Fig. 6 on p. 1912.

After having performed an independent investigation of these data in the Editorial Office, the Editor of *Experimental and Therapeutic Medicine* was able to validate the reader’s concern. In view of the fact that these possible anomalies have been identified in this paper, the Editor has decided that it should be retracted from the Journal on account of a lack of confidence in the presented data. The authors were asked for an explanation to account for these concerns, but the Editorial Office did not receive a reply. The Editor apologizes to the readership for any inconvenience caused.

